# A descriptive, cross-sectional study of postpartum education: midwives’ self-reported knowledge and teaching of postpartum complications in Ghana

**DOI:** 10.1186/s12978-022-01376-z

**Published:** 2022-03-28

**Authors:** Yenupini Joyce Adams, Lynn Sladek

**Affiliations:** 1 Keough School of Global Affairs, University of Notre Dame, 4035 Jenkins Nanovic Halls, Notre Dame, IN 46556 USA; 2 Eck Institute for Global Health, University of Notre Dame, Notre Dame, IN USA; 3 Labor and Delivery, Piedmont Atlanta Hospital, Atlanta, GA USA

**Keywords:** Postpartum, Midwives, Postpartum complications, Discharge education, Maternal mortality, Maternal health

## Abstract

**Background:**

Obstetric complications remain the leading causes of maternal deaths. Since it is not always possible to ascertain which women will develop complications and which women will not, all women who have a baby should be educated about warning signs of complications. In this study, we assessed postpartum education provided by midwives, midwives’ knowledge to teach patients about complications and their skills to manage postpartum complications.

**Methods:**

Descriptive, cross-sectional study of 245 midwives in four hospitals in Tamale, Ghana, using an electronic questionnaire. Data analyzed in Stata 16 software using descriptive, bivariate, and multivariate statistics.

**Results:**

Majority of midwives were female (98%). Mean age of midwives was 32 years. Most midwives spent 6 to 15 min teaching patients on warning signs of complications (61.89%). Mode of discharge education was mostly individual (83.13%). Most midwives reported no reference materials given to patients (66.39%). About 93.45% of midwives strongly agreed or agreed it is their responsibility to teach all patients, regardless of risk factors, about warning signs of complications. However, midwives did not always teach patients about complications. The majority of midwives felt they were knowledgeable or very knowledgeable to teach patients about hemorrhage (95.08%), infection (94.67%), preeclampsia/ eclampsia (90.95%), and hypertension (89.35%). Similarly, most midwives felt they had the skills to manage these same four obstetric complications. Unsurprisingly, most midwives were more likely to always educate their patients about hemorrhage, infection, preeclampsia/ eclampsia, and hypertension—the complications they were more knowledgeable about. Many midwives felt not knowledgeable about and not competent to manage postpartum depression, cardiac events, pulmonary embolism, and venous thrombosis. In the same regard, many midwives did not teach patients about the life-threatening complications they were least knowledgeable about.

**Conclusions:**

Midwives did not always teach patients about complications. Most midwives felt knowledgeable to teach and manage hemorrhage, infection, and preeclampsia/hypertension but not cardiac events, pulmonary embolism, and venous thrombosis. Additional training of midwives on life-threatening complications such as pulmonary embolism and cardiac events is recommended.

**Supplementary Information:**

The online version contains supplementary material available at 10.1186/s12978-022-01376-z.

## Plain language summary

Complications after childbirth are the leading causes of maternal deaths. All women who have a baby should be educated about the warning signs of complications. In this study, 245 midwives from four hospitals in Tamale, Ghana, completed questions that assessed the education they provide to patients on potential complications after childbirth, their knowledge to teach patients about complications, and their skills to manage complications after birth. Midwives reported that patients stay in the hospital for a day or less if they delivered vaginally or for 3 or more days if they delivered by cesarean section. About 62% of midwives spent 6 to 15 min teaching patients on warning signs of complications; 82% provided individual teaching during discharge; and 66% did not provide take-home educational materials to patients. About 93% of midwives either strongly agreed or agreed that it is their responsibility to teach all patients about warning signs of complications. Results indicated that midwives did not always teach patients about potential complications that can occur after childbirth. Midwives more frequently taught patients about severe bleeding, infections, and complications of high blood pressure, and least frequently taught patients about heart-related complications, blood clots and depression after birth. Most midwives felt they were knowledgeable to teach and manage severe bleeding (hemorrhage), infection, and complications related to high blood pressure (preeclampsia/eclampsia) but not heart-related complications (cardiac events), blood clots in lungs (pulmonary embolism), blood clots in deep vein, usually in legs (venous thrombosis), and depression after childbirth.

**Supplementary Information:**

The online version contains supplementary material available at 10.1186/s12978-022-01376-z.

## Background

Despite the world’s maternal mortality ratio (MMR)—defined as the number of maternal deaths per 100,000 live births—declining by 38% between 2000 and 2017, complications resulting from pregnancy and childbirth remain the leading cause of death among women of childbearing age in developing countries [1]. A maternal death is when a woman dies while pregnant or within 42 days of termination of pregnancy, irrespective of the duration and site of the pregnancy, from any cause related to or aggravated by the pregnancy or its management, but not from unintentional or incidental causes [2]. Sub-Saharan Africa accounts for nearly two thirds of all maternal deaths worldwide, and Ghana, located on the western coast of sub-Saharan Africa, has high maternal death rates with an MMR of 308 maternal deaths per 100,000 live births [2]. This MMR is higher than the world MMR of 211 maternal deaths per 100,000 live births and significantly higher than the MMR for high income countries of 11 maternal deaths per 100,000 live births [2]. Globally, countries are working towards meeting the Sustainable Development Goal (SDG) 3 target of reducing the global MMR from 216 to below 70 maternal deaths per 10,000 live births by 2030, with no country having an MMR of more than twice the global average [1, 3]. According to Alkema et al. [4], Ghana must sustain an annual 7.5% MMR reduction between 2016 and 2030 for the world to achieve this ambitious SDG goal. Ghana’s annual rate of MMR reduction between 1990 and 2013 was 1.1%, which is insufficient progress to meet their SDG target of less than twice the global average, that is, less than 140 maternal deaths/100,000 live births [5].

Countries with slow MMR reduction rates should focus their attention and resources on interventions that address the most prevalent causes and timing of maternal deaths within their country or region. Most maternal deaths result from one of seven causes: complications from abortion, embolism, hemorrhage, hypertensive disorders, sepsis, other direct causes, and indirect causes–for example pre-existing conditions excerbated by pregnancy, like cardiomyopathy [6]. In sub-Saharan Africa, the leading causes of maternal deaths from direct obstetric complications are hemorrhage [24.5%], hypertension [16.0%], and sepsis [10.3%] [6]. The timing of maternal deaths is generally classified among one of four timeframes: antepartum (during pregnancy before the onset of labor), intrapartum (during labor up to 24 h post-delivery), postpartum (24 h to 42 days after delivery), or late maternal death (43 days to 1 year after delivery) [5]. In sub-Saharan Africa, over 60% of maternal deaths occur during the postpartum or late maternal death period [5]. Knowing this, targeted interventions should address direct obstetric complications that occur during the postpartum and late postpartum timeframes in an effort to accelerate a reduction in maternal death rates.

Patient education on potential complications by their healthcare provider is an intervention that could improve maternal health outcomes during the critical postpartum time frame for women in Ghana and other countries around the world. Patient education is widely accepted as a low-cost, effective intervention that improves health outcomes [7–9]. In fact, a recent study in Ghana that explored the relationship between education by healthcare providers and a woman’s knowledge and understanding of pre-eclampsia found women who were educated by providers demonstrated higher knowledge and understanding of this leading obstetric complication [10]. Despite knowing this, significant gaps remain in provider’s counseling and education practices for women on obstetric complications.

Quality patient education is imperative when the patient is responsible for their own health care needs. In Ghana, after 48 h from delivery women are expected to independently manage their postpartum care [11]. Fortunately, the causes and associated symptoms of most obstetric complications that occur during the perinatal period are well known and are preventable or manageable when addressed in a timely manner [1, 12]. Patient education that increases knowledge and awareness of the signs and symptoms associated with postpartum complications can lead to a woman’s timely identification and decision to seek treatment of these sometimes-life-threatening complications. This timely identification and decision to seek treatment is of critical importance because the most significant indicator for an adverse maternal outcome is a delay in treatment [13, 14].

Midwives, who attend the majority of births in Ghana, are uniquely positioned to influence the timely identification and treatment of postpartum complications through patient education [11]. In addition to providing primary ante, intra, and postpartum care, midwives are responsible for health counseling and education for their patients [15]. Fortunately, research from Owusu-Addo [16] indicates midwives in Ghana view patient education, particularly patient education surrounding pregnancy, as central to their role as health care providers. Although it is promising that midwives understand this responsibility, it is important to evaluate the quality of postpartum education midwives provide their patients. Likewise, it is important to understand how midwives’ regard their ability or competence to provide general postpartum patient education and postpartum education that discusses the warning signs of obstetric complications in particular. In doing this, we can identify and improve upon gaps that may exist in current postpartum patient education practices.

## Methods

### Aim of study

The purpose of this study was to (1) assess the education provided to patients on potential complications after childbirth self-reported by midwives, and (2) assess midwives’ self-report of their knowledge to teach patients about complications, and their skills to manage postpartum complications.

### Design/setting/participants

This was a descriptive study of midwives who worked in the four main hospitals providing in-patient obstetric services in Tamale, the capital city of the Northern Region of Ghana [17]. Midwives were eligible to participate in the study if they were 18 years and older, and currently working in the four hospitals. Midwives were recruited by convenience sampling. Recruitment and data collection occurred in December 2018. Using precision analysis, a sample size of 243 midwives was needed with a standard deviation of 3 and 0.5 margin of error at a 99% confidence [17].

### Questionnaire

Midwives completed an electronic questionnaire via Qualtrics software. The questions were self-developed by the authors based on review of literature on postpartum education [18–20]. The questionnaire assessed how long women stayed in the hospital, how much time spent teaching patients, mode of teaching, and how often midwives taught patients about hemorrhage, infection, preeclampsia/eclampsia, hypertension, postpartum depression, venous thrombosis, pulmonary embolism, and cardiac event. Midwives were asked whether they felt they had the knowledge to teach patients and skill set to manage the eight complications mentioned above. The questions are published as Additional file 1.

### Recruitment/data collection

One midwife from each of the four hospitals served as research assistants for recruitment and data collection. The questions were pretested by the four midwives for language and context, and minor revisions were made such as changing temperature from degree Celsius to degree Fahrenheit. Research Assistants told midwives at each of their hospitals about the study, and asked of their willingness to participate. Midwives who were willing to participate electronically completed the questions while at the hospital, via a link on a tablet provided to them by the research assistants [17]. The research assistants did not have access to the Qualtrics data. All midwife participants were offered consent prior to participation in the study. Consent was obtained at the beginning of the electronic questionnaire. Midwives, after reading the consent statement, could agree to participate and continue to the questions, or decline participation and exit the questionnaire. No identifying information was collected on the questionnaire. Midwives participated in the study willingly, and were not offered any incentives to participate in the study.

### Ethical approval

The study procedures were reviewed by Kennesaw State University institutional review board and declared as exempt. Approval to conduct the study was also obtained from the Tamale Regional Health Directorate before data collection in the hospitals. In addition, the Department of Research & Development at the Teaching Hospital reviewed and approved the research study.

### Data analysis

Qualtrics data was exported to Stata 16 statistical software for analysis. Descriptive statistics were generated to describe midwife characteristics and summary of results. The outcome variables were knowledge to teach patients, and skill set to manage each of the following complications: hemorrhage, infection, preeclampsia/eclampsia, hypertension, postpartum depression, venous thrombosis, pulmonary embolism, cardiac event. Each of the 8 complications assessed were created into binary variables for each outcome: education about each complication (1—always educate and 0—other), knowledge to teach patients on each complication (1—very knowledgeable/knowledgeable and 0—other), and skill set needed to manage each complication (1—yes and 0—other). The independent variables were hospital, number of deliveries a month, position/rank, qualification/education, length of midwifery training, years of experience, last postpartum care training, last training in infection prevention, last training in postpartum hemorrhage, last training in postpartum assessment of mother, last training in warning signs of complications, last training in postpartum depression, and age. Bivariate logistic regression analysis was conducted to examine possible associations between independent variables and outcome variables. Significant variables (p-value less than 0.05) during bivariate analysis were then put into a multivariate logistic regression model to determine potential predictors of knowledge to teach patients and skill set to manage complications.

## Results

### Midwife characteristics

A total of 246 midwives were approached and started the survey, but 245 completed the questionnaire and were included in data analysis. Out of the 245 midwives, 15 were from Seventh Day Adventist (SDA) hospital, 69 were from Tamale Central hospital, 95 were from Tamale Teaching hospital, and 66 were from Tamale West hospital. The majority of midwives were female (98.0%) with age range from 20 to 59 years. On average, midwives performed about 29 deliveries a month (SD = 21.96). Most midwives were staff midwives (54.29%) and senior staff midwives (23.67%). The majority of midwives either had a diploma in midwifery (46.94%) or a certificate in midwifery (40.41%). Years of midwifery experience ranged from 0 to 20 years, with a mean of about 3.9 years (SD = 3.94). A table presenting the midwife characteristics detailed above has been previously published by the researchers in authors, 2019. About 42% (n = 103) of midwives had never received any in-service training or continuing education in postpartum care. The content of training for those who received some training included: infection prevention (45.71%), postpartum hemorrhage (38.78%), postpartum assessment of mother (33.47%), postpartum warning signs of complications (28.16%), and postpartum depression (8.57%).

### Education provided to patients on potential complications after childbirth

Midwives mentioned that most women after a vaginal delivery spend either one day (n = 135, 55.33%) or less than a day/24 h (n = 83, 34.02%) in the hospital before discharge. For women who delivered via cesarean section, most of them spend three days (n = 173, 70.90%) or four or more days (n = 67, 27.46%) in the hospital before discharge. Since most patients in the setting deliver vaginally and leave the hospital in less than 24 h, quality patient education is important because patients have to be responsible for their own health shortly after birth. Many midwives (n = 96, 39.51%) reported they spend 15–30 min at discharge educating patients on all necessary topics/information. In terms of specific education on warning signs of complications, most midwives either spend 11–15 min (n = 76, 31.15%) or 6–10 min (n = 75, 30.74%) teaching patients on warning signs of complications. Approximately 83% (n = 202) of discharge education is on an individual basis while about 17% occur as group teaching (n = 41). Most midwives indicated that patients are not provided with any reference materials or educational handouts to take home on warning signs of complications (n = 162, 66.39%). See Table 1 for detailed results of patient education after childbirth.

**Table 1:** Summary of patient education after childbirth by midwives

*Time spent teaching patient on all necessary information N = 244*		
10 min or less	32	13.17%
10–15 min	64	**26.34%**
15–30 min	96	**39.51%**
30 min – 1 h	49	20.16%
More than 1 h	2	0.82%
*Time spent teaching patient on warning signs of complications N = 244*		
5 min of less	16	6.56%
6–10 min	75	**30.74%**
11–15 min	76	**31.15%**
16–20 min	26	10.66%
21–30 min	23	9.43%
30 or more minutes	28	11.48%
*Mode of discharge education N = 243*		
Group teaching	41	16.87%
Individual teaching	202	**83.13%**
*Educational handouts/take-home reference provided N = 244*		
No	162	**66.39%**
Yes	82	33.61%

Patient education on potential postpartum complications					
Complication	Always	Most of the time	Sometimes	Never	Only if relevant
Hemorrhage	136 **(55.74%)**	69 (28.28%)	28 (11.48%)	2 (0.82%)	9 (3.69%)
Infection	124 **(50.82%)**	82 (33.61%)	27 (11.07%)	4 (1.64%)	7 (2.87%)
Preeclampsia/Eclampsia	104 **(42.62%)**	74 (30.33%)	31 (12.70%)	4 (1.64%)	31 (12.70%)
Hypertension	82 **(34.17%)**	79 (32.92%)	36 (15.00%)	4 (1.67%)	39 (16.25%)
Postpartum depression	36 **(14.81%)**	49 (20.16%)	65 (26.75%)	17 (7.00%)	76 **(31.28%)**
Venous thrombosis	25 **(10.25%)**	37 (15.16%)	67 (27.46%)	41 (16.80%)	74 **(30.33%)**
Pulmonary embolism	19 **(7.79%)**	37 (15.16%)	60 (24.59%)	45 (18.44%)	83 **(34.02%)**
Cardiac event	22 **(9.09%)**	34 (14.05%)	64 (26.45%)	37 (15.29%)	85 **(35.12%)**

Bold values indicate to help readers visually see the higher percentages

Midwives were asked how often they teach patients about the most common postpartum complications using a scale from “never” to “always”. About 136 (55.74%) midwives reported they always teach patients about hemorrhage, 124 (50.82%) reported they always teach about infection, 104 (42.62%) reported they always teach about preeclampsia/eclampsia, 82 (34.17%) reported they always teach about hypertension, 36 (14.81%) reported they always teach about postpartum depression, 25 (10.25%) reported they always teach about venous thrombosis, 19 (7.79%) reported they always teach about pulmonary embolism, and 22 (9.09%) reported they always teach about cardiac event. More than 30% of midwives said they teach patients about postpartum depression, venous thrombosis, pulmonary embolism, and cardiac event “only if relevant”. Table 1 presents details about midwives’ teaching on the various complications. When asked about responsibility to teach all patients about warning signs of complications, 56.15% (n = 137) of midwives strongly agreed, 37.30% (n = 91) agreed, and less than 1.00% (n = 2) strongly disagreed that it is their responsibility to teach all patients, regardless of risk factors, about warning signs of complications. A few midwives said patients should only be educated when relevant (5.74%, n = 14).

### Midwives’ perceptions: knowledge to teach patients and skills to manage complications

Midwives were asked whether they felt they have the knowledge to teach patients on the most common postpartum complications. The percentage of midwives who felt they were either knowledgeable or very knowledgeable to teach patients about complications were: hemorrhage (95.08%), infection (94.67%), preeclampsia/eclampsia (90.95%), hypertension (89.35%), postpartum depression (54.5%), venous thrombosis (37.86%), pulmonary embolism (34.16%), and cardiac event (33.2%). See Table 2 for more detailed results. The variable hospital was significantly associated with knowledge to teach hemorrhage, infection, preeclampsia/eclampsia, and hypertension (see Table 3). Midwives in the three government hospitals were about two times more likely to teach these four complications than midwives in the mission hospital. Number of deliveries per month was positively associated with knowledge to teach infection and preeclampsia. The odds of being knowledgeable to teach postpartum depression was more than five times in midwives who received in-service or continuing education in postpartum depression than midwives who did not. The odds of being knowledgeable to teach about venous thrombosis was about 1.3 times more likely in senior and principal midwifery officers than in midwives in lower positions. The odds of being knowledgeable to teach about venous thrombosis was also about two times more likely in midwives whose length of midwifery training was 2–3 years than in midwives with 0–1 years of midwifery education (Table 3).

**Table 2:** Midwives’ perceptions of knowledge and skill set to teach and manage postpartum complications

Knowledge to teach patients					
Complication	Very knowledgeable	Knowledgeable	Somewhat knowledgeable	Not knowledgeable	Feel not necessary
Hemorrhage	94 **(38.52%)**	138 **(56.56%)**	12 (4.92%)	–	–
Infection	101 **(41.39%)**	130 **(53.28%)**	13 (5.33%)	–	–
Preeclampsia/eclampsia	83 **(34.16%)**	138 **(56.79%)**	21 (8.64%)	1 (0.41%)	
hypertension	74 **(30.33%)**	144 **(59.02%)**	25 (10.25%)	1 (0.41%)	–
Postpartum depression	35 (14.34%)	98 **(40.16%)**	87 **(35.66%)**	21 **(8.61%)**	3 (1.23%)
Venous thrombosis	27 (11.11%)	65 **(26.75%)**	110 **(45.27%)**	38 **(15.64%)**	3 (1.23%)
Pulmonary embolism	20 (8.23%)	63 **(25.93%)**	112 **(46.09%)**	44 **(18.11%)**	4 (1.65%)
Cardiac event	16 (6.56%)	65 **(26.64%)**	112 **(45.90%)**	49 **(20.08%)**	2 (0.82%)

Skill set to manage complications				
Complication	Yes	Somewhat	No	Don’t know
Hemorrhage	221 **(90.57%)**	18 (7.38%)	4 (1.64%)	1 (0.41%)
Infection	220 **(90.16%)**	16 (6.56%)	5 (2.05%)	3 (1.23%)
Preeclampsia/eclampsia	215 **(88.11%)**	20 (8.20%)	8 (3.28%)	1 (0.41%)
Hypertension	206 **(84.77%)**	25 (10.29%)	11 (4.53%)	1 (0.41%)
Postpartum depression	84 **(34.43%)**	84 **(34.43%)**	69 **(28.28%)**	7 (2.87%)
Venous thrombosis	58 (23.77%)	88 **(36.07%)**	87 **(35.66%)**	11 (4.51%)
Pulmonary embolism	51 (20.90%)	91 **(37.30%)**	90 **(36.89%)**	12 (4.92%)
Cardiac event	41 (16.80%)	94 **(38.52%)**	95 **(38.93%)**	14 (5.74%)

Bold values indicate to help readers visually see the higher percentages

**Table 3:** Results of multivariate analysis of variables that were significant in bivariate analysis

	Midwives’ perceived knowledge to teach patients about complications p-value (odds ratio)							
Independent variable	Hemorrhage	Infection	Preeclampsia / eclampsia	Hypertension	Postpartum depression	Venous thrombosis	Pulmonary embolism	Cardiac event
Hospital	*0.02* (2.19)	*0.04* (1.85)	*0.00* (2.19)	*0.01* (2.02)				0.92 (0.98)
# of deliveries per month	0.06 (1.06)	*0.04* (1.06)	*0.04* (1.04)					
Position / rank						*0.04* (1.28)		0.25 (1.16)
Qualification / education							0.81 (1.07)	0.76 (1.09)
Length of midwifery training					0.37 (1.23)	*0.01* (1.91)	0.08 (1.83)	0.98 (1.96)
Age				0.05 (1.09)				
Last training postpartum hemorrhage				0.52 (1.55)				
Last training postpartum assessment of mother				0.66 (1.39)				
Last training postpartum depression					*0.01* (5.21)			

	Midwives’ perceived skill set to manage complications p-value (odds ratio)							
Independent variable	Hemorrhage	Infection	Preeclampsia / eclampsia	Hypertension	Postpartum depression	Venous thrombosis	Pulmonary embolism	Cardiac event
Hospital	*0.01* (1.93)	*0.02* (1.78)	*0.01* (1.93)	*0.00* (1.91)				
# of deliveries per month	*0.01* (1.06)	*0.01* (1.06)	*0.01* (1.06)					
Qualification / education					*0.02* (2.01)	0.09 (1.47)	0.57 (1.21)	0.16 (1.44)
Length of midwifery training					0.69 (0.87)		0.15 (1.80)	
Years of experience			0.95 (1.01)	0.66 (1.05)				
Age		0.09 (1.09)	0.26 (1.07)	0.29 (1.06)				
Last training in postpartum care	0.11 (2.67)			0.08 (2.56)				
Last training infection prevention	0.50 (0.55)	0.36 (1.83)	0.22 (2.15)	0.88 (0.90)				
Last training postpartum hemorrhage	0.65 (1.56)	0.45 (1.73)	0.61 (0.70)	0.95 (0.95)				
Last training postpartum assessment of mother				0.58 (0.67)				
Last training postpartum warning signs of complications	0.61 (1.70)		0.22 (2.72)					
Last training postpartum depression						0.04 (2.70)	0.02 (3.03)	0.01 (3.69)

Significant p-values in italics font

Likewise, midwives were asked whether they had the skill set needed to manage these complications. The percentage of midwives who felt they had the skill set to manage complications were: hemorrhage (90.57%), infection (90.16%), preeclampsia/eclampsia (88.11%), hypertension (84.77%), postpartum depression (34.43%), venous thrombosis (23.77%), pulmonary embolism (20.90%), and cardiac event (16.80%). See Table 2 for more detailed results. Midwives in the government hospitals were about two times more likely to have the skill set to manage hemorrhage, infection, preeclampsia, and hypertension than midwives in the mission hospital (Table 3). Midwives who had a diploma or bachelors in midwifery were two times more likely to have the skill to manage postpartum depression than midwives with a certificate in midwifery. Number of deliveries per month was positively associated with skill to manage hemorrhage, infection, and preeclampsia. Midwives who received in-service training on postpartum depression were three times more likely to say they had the skill to manage venous thrombosis, pulmonary embolism, and cardiac event (Table 3).

## Discussion

Results from this study reveal two significant findings. First, midwives can improve both the content and the delivery of educating patients on warning signs of obstetric complications. And second, there appears to be a relationship between midwives’ own knowledge of specific obstetric complications—for example their knowledge of how to recognize and manage hemorrhage or infection—and their likelihood of educating patients on that specific complication. This not only makes sense but provides valuable insight into how to target interventions to improve postpartum education practices. This finding suggests that improving midwives’ knowledge of and skills to manage obstetric complications would likely increase postpartum patient education practices.

Another important finding from our study is the disconnect between midwives’ understanding of their responsibility to provide postpartum education and midwives’ patient education practices. The overwhelming majority of midwives (93.45%) either agreed or strongly agreed that it was their responsibility to teach all patients, regardless of risk factors, about warning signs of complications. Yet, the most common complication, hemorrhage, received only a 55.74% “always discussed” response rate. Of the seven remaining obstetric complications studied, only infection achieved a better than half “always discussed” response rate from the midwives. Follow-up focus group discussions or qualitative interviews are needed to understand why midwives may not always teach about the most common complications accountable for maternal deaths in the setting. Furthermore, midwives were less likely to educate patients about less common but life-threatening complications such as depression, venous thrombosis, pulmonary embolism, or cardiac events. While not the most common complications in the setting, postpartum depression, pulmonary embolism, and cardiac issues can cause significant morbidity and can be fatal if not recognized and managed in a timely fashion. We argue that while emphasis should be placed on the most common complications, less common but deadly complications such as pulmonary embolism and cardiac issues should also be discussed. When women are adequately educated about complications and can recognize obstetric warning signs, they are better equipped to make the decision to seek timely treatment for these potentially life-threatening complications [21, 22].

Our study of midwives’ knowledge of obstetric complications mirrored research findings that examine women’s knowledge of obstetric complications in low-to-middle-income countries (LMIC) with high maternal mortality ratios: women are most knowledgeable about hemorrhage, infection, and symptoms of uncontrolled hypertension but lack knowledge about other obstetric complications [23, 24]. This finding reinforces the need for midwives to increase their knowledge and their teaching of all obstetric complications to empower women to make timely, informed decisions on when to seek medical care. In another study on midwives’ knowledge of post-birth warning signs, results indicated that most midwives could identify warning signs of hemorrhage, high blood pressure and infection, but not warning signs of postpartum depression, pulmonary embolism, and cardiac events [17]. While it is noteworthy midwives and researchers alike are correctly prioritizing education and information regarding obstetric warning signs that mirror the leading causes of maternal death—hemorrhage, hypertension and its complications, and infection—there remains the opportunity to improve education and research regarding all obstetric warning signs.

Our study also found midwives can improve their delivery of postpartum education. Most education on obstetric complications lasted 6–15 min (61.89%); 83.13% of education was conducted on an individual basis, and 66.39% of education was verbal only, with no reference materials given to patients. Quality postpartum education can be difficult to provide because of the large amount of information that needs to be shared [19, 20] and the short amount of time a woman is hospitalized after birth. We speculate that the constraints of abundance of material to cover in a short amount of time for quality postpartum education prove true for midwives in Ghana. For these reasons, reference materials or educational handouts can help bridge the gap between the constraints of large content and limited time [25]. Patient handouts or reference materials that are context appropriate and at literacy levels of patients are recommended. In addition, we recommend a standardized patient education checklist and content materials that midwives can use to provide quality postpartum education to every patient. Given that midwives felt less knowledgeable to teach and less capable to manage postpartum depression, venous thrombosis, pulmonary embolism, and cardiac events, we recommend midwives receive additional training on these life-threatening complications.

### Limitations

Our study adds to the limited literature on postpartum education of obstetric complications, and the importance of this research cannot be overstated. Most maternal deaths occur in the postpartum period from causes that are well known and preventable when recognized and treated early; yet, very little research focuses on patient education practices during this time frame. While our study had a good sample size with very few missing data, it is not without limitations. First, midwives were recruited through convenience sampling by colleague midwives, thus, results may not represent the entire population. Second, due to the educational level of midwives in the study context, results may not be generalizable to contexts in which midwives have graduate degrees or are educated as advanced practice registered nurses. Further, since this was a descriptive study, we can only report associations in the analysis of potential predictors. Finally, data analysis is from self-report data, and we did not conduct analysis of hospital data to incorporate individual hospital differences.

## Conclusions

This study provides unique insights into how to improve midwives’ postpartum education practices on obstetric warning signs. Interventions that are focused on increasing midwives’ knowledge to identify and treat obstetric complications offers the potential to improve midwives' education practices regarding the same. Because obstetric complications are the leading causes of maternal deaths and time is of the essence in identifying and seeking treatment for these complications, it is important that midwives consistently educate patients about obstetric warning signs. To more effectively do this, hospitals should provide initial and ongoing education and training to increase midwives' knowledge to recognize and manage all obstetric complications. In doing this, midwives can improve their knowledge and their skills of obstetric complications while also empowering patients, through postpartum education of obstetric warning signs, to better recognize when to make the timely, life-saving decision to seek medical treatment.

## Supplementary Information

**Additional file 1. **Postpartum patient education questions.

## Data Availability

The dataset analyzed for the current study is available from the corresponding author on reasonable request.

## Abbreviations

MMR: Maternal mortality ratio
WHO: World Health Organization
SDG: Sustainable development goal
UN: United Nations
GSS: Ghana Statistical Service
GHS: Ghana Health Service
