# Supplementary material for: A descriptive, cross-sectional study of postpartum education: midwives’ self-reported knowledge and teaching of postpartum complications in Ghana
Source: Reprod Health. 2022 Mar 28;19:77. doi: 10.1186/s12978-022-01376-z (PMC8962571; doi:10.1186/s12978-022-01376-z)
Supplement: Supplementary file 1 — Additional file 1. Postpartum patient education questions. [file 12978_2022_1376_MOESM1_ESM.docx]

**Additional File**

**Additional file 1. Postpartum Patient Education Questions**

| How long do women stay before discharge if vaginal delivery?  Less than 24 hours/same day  1 day  2 days  3 days  4 days or more |
| --- |
| How long do women stay before discharge if cesarean delivery?  Less than 24 hours/same day  1 day  2 days  3 days  4 days or more |
| How much time do you spend at discharge teaching the patient on all necessary topics/or information they need to know?  10min or less  10-15min  15-30min  30min-1hour |
| How much time do you spend at discharge teaching the patient on warning signs of complications alone?  5min or less  6-1 0mm 11-15mm  16-20mm 21-30mm  30min or more |
| How is discharge teaching performed?  Individual teaching  Group teaching |
| Are any handouts provided to the patient to take home for reference on warning signs of complications?  Yes  No |
| How often do you teach the following topics to patients?  Never Sometimes Only if relevant Most of the time Always  Hemorrhage  Infection  preeclampsia/eclampsia  hypertension  postpartum depression  venous thrombosis  pulmonary embolism  cardiac event |
| It is the responsibility of the midwife to teach all patients, even those with no risk factors, about warning signs of complications  Strongly agree  Agree  Only when Relevant  Disagree  Strongly Disagree |
| Do you feel you have the knowledge to teach patients on the following complications?  Very Knowledgeable Knowledgeable Somewhat Knowledgeable Not Knowledgeable Feel not necessary  Hemorrhage  Infection  preeclampsia/eclampsia  hypertension  postpartum depression  venous thrombosis  pulmonary embolism  cardiac event |
| Do you feel you have the skill set needed to manage these complications?  Yes No Somewhat Don’t Know  Hemorrhage  Infection  preeclampsia/eclampsia  hypertension  postpartum depression  venous thrombosis  pulmonary embolism  cardiac event |
